# The importance of professional values from clinical nurses’ perspective in hospitals of a medical university in Iran

**DOI:** 10.1186/s12910-017-0178-9

**Published:** 2017-03-01

**Authors:** Batool Poorchangizi, Jamileh Farokhzadian, Abbas Abbaszadeh, Moghaddameh Mirzaee, Fariba Borhani

**Affiliations:** 1 Nursing Research Center, Kerman University of Medical Sciences, Kerman, Iran; 2 Department of Community Health Nursing, School of Nursing and Midwifery, Nursing Research Center, Kerman University of Medical Sciences, PO Box: 7716913555, Haft-bagh Highway, Kerman, Iran; 3 Department of Nursing, Nursing & Midwifery School, Shahid Beheshti University of Medical Sciences, Tehran, Iran; 4 Department of Biostatistics and Epidemiology, School of Public Health, Kerman University of Medical Sciences, Kerman, Iran; 5 Department of Nursing Ethics, Medical Ethics and Law Research Center, Shahid Beheshti University of Medical Sciences, Niyayesh Complex, Niyayesh Cross-Section, Vali Asr St, PO Box: 1985717443, Tehran, Iran

**Keywords:** Ethics, Nursing, Professional values, Nursing values

## Abstract

**Background:**

Today, nurses are required to have knowledge and awareness concerning professional values as standards to provide safe and high-quality ethical care. Nurses’ perspective on professional values affects decision-making and patient care. Therefore, the present study aimed to investigate the importance of professional values from clinical nurses’ perspective.

**Methods:**

The present cross-sectional study was conducted in 2016 in four educational hospitals of Kerman University of Medical Sciences, Iran. Data were collected via the Persian version of Nursing Professional Values Scale-Revised (NPVS-R) by Weis and Schank. Sampling was conducted through the use of stratified random sampling method and 250 clinical nurses participated in the study.

**Results:**

Results indicated that the total score of the nurses’ professional values was high. (102.57 ± 11.94). From nurses’ perspective items such as “Maintaining confidentiality of patients” and “Safeguarding patients’ right to privacy” had more importance; however, “Recognizing role of professional nursing associations in shaping healthcare policy” and “Participating in nursing research and/or implementing research findings appropriate to practice had less importance. A statistically significant relationship was observed between NPVS-R mean scores and nurses’ age, work experience as well as participation in professional ethical training (*P* < 0.05).

**Conclusions:**

Although the total score related to the clinical nurses’ perspective on professional values was high, the importance of certain values was at a lower level. Owing to the emerging ethical challenges, it is indispensable to design educational programs in order to improve nurses’ awareness and understanding of the comprehensive importance of professional values. Furthermore, it is recommended that mixed methods studies should be conducted in order to design an instrument to evaluate the use of values in nursing practice.

## Background

Any professional group has primary standards called professional values. These values are considered as the guideline and motivation of professional behavior for the members of a certain profession [1]. According to Weis and Schank, professional values are standards for action that are accepted by professional groups and individuals, and are used to evaluate the integrity of the individual or organization. In addition, professional values are necessary to reinforce individuals’ the professional identity and performance [2]. Professional values are rooted in personal values, which are influenced by family, culture, environment, religion, and ethnicity. The process of acquisition such values is gradual and evolutionary and occurs throughout an individual’s lifetime [3].

Nurses, as the largest health care group, have well-known and important professional values. The use of these values in nursing practice increased the quality of patients care, nurses’ occupational satisfaction, their retention in nursing and commitment to the organization [4, 5]. Professional values are a source to promote nurses’ ethical competencies in clinical settings and dealing with ethical concerns in the present era [6]. Most nurses are aware of ethical issues, but they do not use them in their clinical practice. Furthermore, in many cases, they lack sufficient power and support to demonstrate their reaction in this regard [7] or they are not aware of its importance [8]. Thus, professional values are a solution to the current problems in nursing profession [6]. Today, globalization, migration, nursing shortage, new diseases, ageing population, and demand for high-quality care are complicated issues that result in ethical problems for nurses [9]. Therefore, they are expected to be aware of professional values and apply them to their decision makings while dealing with such ethical problems [10, 11].

Values are acquisitive; this means they learned either directly or indirectly by observing others’ behavior [12, 13]. Following academic education, development of professional values in nurses is mainly influenced by experts in the profession, colleagues, patient care situations, and organizational values [14, 15].

Various studies on professional values in different countries have reported that the difference in professional values lies in their prioritization nor in their nature. These researches have highlighted that the difference in priorities might be owing to the cultural, social, economic, and religious situations [10, 16]. For instance, Rassin’s study in Israel, demonstrated that priority values were different among the nursing students of different ethnic groups [3]. The study conducted by Shahriari et al. entitled “Ethical values perceived by nurses” indicated that Iranian nurses, due to their religious beliefs, placed more emphasis on preserving patients’ dignity [17]. Two other studies showed that nurses’ knowledge of professional values and how such values influence their behavior is an essential component of nursing care [2, 18]. Moreover, some other studies represented that nurses have low knowledge and awareness of professional values do not use them in action to shape their ethical thinking and rely merely on personal experiences or organizational culture as the basis of their ethical responsibility and commitment [19, 20]. Therefore, it is important to obtain the basic information on nurses’ awareness of their professional values in different clinical environments. Furthermore, examining the nurses’ perspective on the importance of professional values in different environments and cultures would help healthcare managers to perceive the differences in professional individuals’ value systems, thereby creating an appropriate working environment for nurses [21]. Moreover, the complexity pertaining to advanced healthcare increases the need to conduct research and provide the basic information for education in the field of professional values and ethics. The present study was aimed to investigate the importance of professional values from clinical nurses’ perspective.

## Methods

### Aim

The aim of this study was to explore the importance of professional values from nurses’ perspective.

### Design and setting

The present cross-sectional study was conducted from September to December, 2016 in four hospitals affiliated with Kerman University of Medical Sciences (KUMS), the largest city in the southeast of Iran.

### Sample/participants

The target population included all nurses employed at the time of data collection (n =1113). Using the sample size formula, 260 clinical nurses were invited to participate in the study. The inclusion criteria included the head nurses and nurses involved in the direct patient care with academic degree and working experience of more than one year. Incomplete questionnaire was considered the exclusion criterion. The samples were selected from the clinical wards using stratified random sampling method proportionate to the number of nurses per hospital. Finally, 250 participants completed the questionnaires (response rate 96%). Therefore, from the populations of 279, 391, 313 and 130 nurses in the four hospitals, 64, 93, 73, and 30 nurses enrolled, respectively.

### Data collection

Data collection was performed using a two-section questionnaire. The first section collected the participants' demographic characteristics including gender, marital status, level of education, ethnicity, type of employment, amount of income, and participation in professional ethical training. The second section was Weis and Schank’s Nursing Professional Values Scale-Revised (NPVS-R). The NPVS-R is the only known instrument for measuring professional nursing values. In order to develop the professional values scale, Weis and Schank used the ANA Code of Ethics as well as the studies related to the values and professional value development in nurses [2]. The NPVS-R has also recently been translated and validated into different languages including Chinese [22], Spanish [23], Turkish [24], and Korean [25]. It has been also used in several studies in Iran, and its reliability and validity have been approved [26, 27]. In the present study, the Persian version of the NPVS-R translated by Hosseini et al. was used. To ensure reliability, a pilot study was conducted on 20 nurses in which Cronbach’s alpha coefficient was calculated as 0.90.

The NPVS-R consisted of 26 items in five dimensions of caring (nine items), activism (five items), professionalism (four items), trust (five items) and justice (three items). The NPVS-R used a 5-point Likert-like scale ranging from 1 to 5, with score 1 as not important, score 2 somewhat important, score 3 important, score 4 very important and score 5 the most important. The possible range of scores was between 26 and 130. The more importance an individual ascribes to a scale item was reflected in a higher total score, indicating greater congruency with the professional values measured by the NPVS-R [2]. Additionally, the scores below 43, between 43 and 86, and above 86 indicated low, medium, and high importance, respectively.

For data collection, the first researcher referred to the intended wards in different shift works. After explaining how to fill the questionnaire as well as defining the research objectives, the researcher distributed the Persian questionnaires among the participants and asked them to specify their knowledge concerning the importance of professional values. To achieve the same perception of the questionnaire’s items and eliminate any kind of ambiguity concerning answering the questions for all the nurses, the researcher provided the participants with necessary explanations and then collected the questionnaires with maintaining confidentiality.

### Statistical analysis

Data were analyzed via SPSS version 19 using descriptive statistics (frequency, percentage, mean and standard deviation) and inferential statistics (independent samples, t- test, analysis of variance (ANOVA), Mann-Whitney, Kruskal-Wallis and Pearson’s correlation coefficient). Level of significance was considered *P* < 0.05.

## Results

Results showed that the mean age was 32.7 ± 7.37 years and the mean working experience of the participants was 6.64 ± 9.17 years. The majority of the participants were female (90%), married (75.6%), and had Bachelor’s degree (98.4%), as well as were of Persian ethnicity (98.4%). More than half of the participants (54.8%) had an amount of income higher than $440.66. In addition, most of participants were contract recruiters (36.8%) and almost 62.4% participated in professional ethical training (Table 1).

**Table 1:** Nurses’ demographic information and its relationship with NPVS-R mean scores (*n* = 250)

Variable		n	%	Mean ± SD	Test statistic	*P*
Gender	Female	225	90%	103.03 ± 1.18	t = 1.79	0.09
	Male	25	10%	98.52 ± 1.21		
Marital status	Married	189	75.6%	103.2 ± 1.16	t = 1.44	0.15
	Single	61	24.4%	100.66 ± 1.27		
Ethnicity	Persian	246	98.4%	102.58 ± 1.17	Z = 0.24	0.98
	Non Persian	4	1.6%	102.25 ± 2.26		
Level of education	Bachelor’s degree	233	93.2%	102.73 ± 11.98	t = 0.99	0.32
	Master’s degree	17	6.8%	105.35 ± 11.42		
Participation in professional ethical training	Yes	156	62.4%	104.26 ± 1.18	t = 2.91	0/004^*^
	No	94	37.6%	99.77 ± 1.17		
Amount of income	$ 264.39- 323.15	8	3.2%	99.75 ± 1.47	F = 5.08	0.07
	$ 352.53- 440.66	105	42%	99.88 ± 1.28		
	more than $ 440.66	137	54.8%	104.73 ± 1.07		
Type of employment	Hired	47	18.8%	106 ± 10.9	*χ* ^2^ = 9.32	0.054
	Contract recruiters^a^	92	36.8%	103 ± 11.84		
	Contract recruiters^b^	66	26.4%	99.87 ± 11.59		
	Committed^c^	45	18%	99.31 ± 12.76		

^a^annually contracted with payment similar to hired nurses; ^b^annually contracted with payment less than hired nurses; ^c^it is obligatory to work for government for 2 years at a lower rate of payment. ^*^Significant (*p* < 0.05)

The mean total score of the importance of professional values from the nurses’ perspectives was high (102.57 ± 11.94). Table 2 shows that according to the five-point Likert scale, each item falls in the range of important to very important. Based on the mean scores, the most important values from the nurses’ perspective were “Maintaining confidentiality of patients” (4.54 ± 0.64) and “Safeguarding patients’ right to privacy” (4.38 ± 0.68) from caring dimension, “Assuming responsibility for meeting the health needs of culturally diverse population” (4.34 ± 0.70) from trust dimension, and “Accepting responsibility accountability for their own practice” (4.33 ± 0.68) from justice dimension, respectively. While, the least important values were “Recognizing role of professional nursing associations in shaping healthcare policy” (3.44 ± 0.96), “Participating in nursing research and/or implementing research findings appropriate to practice (3.44 ± 0.91), and “Participating in public policy decisions affecting distribution of resources” (3.53 ± 0.96) concerning activism dimension, and “Participating in peer review” (3.53 ± 0.88) concerning professionalism dimension, respectively (Table 2).

**Table 2:** NPVS-R item rank and mean scores in nurses

Dimension	Item	Rank	Mean Score	SD
Justice	Engage in ongoing self- evaluation	19	3.71	0.80
	Request consultation/collaboration when unable to meet patient needs	12	4.00	0.69
	Seek additional education to update knowledge and skills	8	4.15	0.79
	Accept responsibility and accountability for own practice	4	4.33	0.68
	Maintain competency in area of practice	5	4.30	0.72
Trust	Protect health and safety of the public	11	4.03	0.73
	Promote equitable access to nursing and healthcare	13	3.99	0.78
	Assume responsibility for meeting health needs of Culturally diverse population	3	4.34	0.70
Professionalism	Participate in peer review	23	3.53	0.88
	Establish standards as a guide for nursing practice	18	3.73	0.83
	Promote and maintain standards where planned learning activities for students take place	20	3.71	0.91
	Initiate actions to improve environments of practice	14	3.91	0.84
Activism	Participate in public policy decisions affecting distribution of resources	24	3.53	0.96
	Advance the profession through active involvement in health-related activities	16	3.85	0.79
	Recognize role of professional nursing associations in shaping healthcare policy	26	3.44	0.96
	Participate in nursing research and/or implement research findings appropriate to practice	25	3.44	0.91
	Participate in activities of professional nursing associations	22	3.58	1.56
Caring	Protect moral and legal rights of patients	6	4.22	0.75
	Refuse to participate in care if in ethical opposition to own professional values	9	4.15	0.87
	Act as a patient advocate	10	4.08	0.79
	Provide care without prejudice to patients of varying Lifestyles	15	3.89	0.86
	Safeguard patient’s right to privacy	2	4.38	0.68
	Confront practitioners with questionable or inappropriate practice	17	3.81	0.90
	Protect rights of participants in research	21	3.68	0.83
	Practice guided by principles of fidelity and respect for person	7	4.16	0.71
	Maintain confidentiality of patient	1	4.54	0.64
	Total		102.57	11.94

The results of Spearman’s correlation coefficient test showed the poorly significant relationship between the NPVS-R mean scores and age (r = 0.162, *P* = 0.010) and working experience (r = 0.19. *P* = 0.003). In other words, the older and more experienced nurses obtained higher scores of professional values. Furthermore, as Table 1 shows, the NPVS-R scores had a significant relationship with participating in professional ethical training (t = 1.40, *P* = 0.004). No significant relationship was observed between the NPVS-R scores and other demographic variables such as marital status, level of education, ethnicity, amount of income, and type of employment (*P* > 0.05).

## Discussion

The present study was aimed to investigate the importance of the professional values from nurses’ perspective. The obtained results showed that the mean total score of the importance of professional values was high. Similar to the present study, Clark [15], Gallegos and Sortedahl [28], and Schank and Weis [29] reported high score for nurses’ professional values to justify this high score. The American Association of Colleges of Nursing reported that the majority of individuals entering nursing profession have accepted that professional values are highly important in nursing, although they might not apply all the values equally in practice [30]. One other reason for such high scores may be the questionnaire items, which the nurses had more tendency to choose “very important” and “most important" options, ultimately leading to exaggerated scores.

In the present study, the most important professional values according to the nurses were “Maintaining confidentiality of patients”, “Safeguarding patients’ right to privacy”, “Assuming responsibility for meeting health needs of culturally diverse population”, and “Accepting responsibility and accountability for their own practice”, respectively. Consistent with the present study results, these values were identified as the most important nursing professional values in other studies conducted by Gallegos and Sortedahl [28], Clark [15], Schank and Weis [29], as well as Shahriari et al. One reason for this might be the fact that nurses mainly consider values that are directly related to nursing care and nurses' daily clinical work [15]. Another reason might be people’s high expectations from nurses concerning such values so that Leners et al. reported this case in their study. Furthermore, they believed that these values might inherently exist in individuals with tendency towards nursing profession [13].

In the present study, certain values were less important based on the nurses’ perspective, such as “Recognizing role of professional nursing associations in shaping health care policy”, “Participating in nursing research and/or implementing research findings appropriate to practice”, “Participating in public policy decisions affecting distribution of resources”, and “Participating in peer review”, respectively. In several studies [15, 28, 29], these items are among the least important values, but the priority and ranking of these values are different with the present study. Probably, there are several factors that nurses not willing to accept all the values equally such as personal beliefs and attitudes [18], conditions and factors pertaining to the working environment, working pressures and organizational culture, level of organizational support [14, 31], awareness, educational programs, and level of motivation and interest [20] can affect the prioritization of professional values. Perhaps, another reason for the low importance of the foregoing values might be insufficient emphasis on emotional learning by university educators and tutors at the time of the formation of professional values in the affective domain of learning. If these values are not encouraged or noticed during academic education, they might fully not accepted [15]. Leners et al. stated that the above-mentioned values are related to the collaborative and social activities in nursing and healthcare organizations. Therefore, there is low awareness regarding their direct relationship with the promotion of nursing profession [13].

Regarding the low importance “Recognizing role of professional nursing associations in shaping healthcare policy”, from the nurses’ perspective, Schank and Weis stated that the activities related to these associations are out of working hours, and diminish the caring role. Moreover, they believed that it is optional to participate and perform activities in nursing associations. Therefore, nurses have low awareness of the role of professional nursing associations [29]. It is suggested that these values be introduced to nurses and the conditions are provided to participate in these activities in order to contribute to promote the nursing profession.

The reasons for the low importance of values such as “Participation in the nursing research and the clinical application of the findings” in Iran are probably the unfavorable attitude towards the application of the research and evidence-based practice, and also low self-efficacy in this field, as well as poor information literacy skills such as searching sources of information, organizing information in databases, information retrieval skills, and evaluating evidence, which were published in two articles by Farokhzadian et al. [32, 33].

Regarding values such as “Participating in peer review” and “Participating in public policy decisions affecting distribution of resources”, it can be speculated that the organizational structure in Iran is such that these activities are among the duties of managers in hospitals. Performance evaluation is conducted by managers and the staff do not involve in peer evaluation. Hence, organizational structures need to be adjusted in accordance with nursing professional values.

In the present study, age had a positively and poorly significant relationship with the scores of professional values. These results are consistent with Kubsch et al. study [31], while in contrast with Shahriari et al., study that the nurses grow older had the lower score of professional values [34]. Probably, it can be said that the increase in the age led to more stable personality as well as higher adaptability with problems. Furthermore, older age by repeating the experiences for nurses reinforce professional values [31].

In this study, there was a positively and poorly significant relationship between the work experience and score of professional values. Result of the present study is consistent with Gallegos and Sortedahl’s study. In their study, the low scores were initiated with 3 to 10 years of work experience; indicate that nurses moving toward competent stage face challenges and formative experiences that reflected in scores of professional values [28]. In Clark’s study, consistent with our study, the scores of professional values were enhanced with the increase in the nurses’ experience [15]. In contrast to the present study, Leduc and Kotzer demonstrated that there was no significant difference between the working experience and professional values [35]. Such differences might be due to the existence of various age groups, clinical environments or working conditions.

The present study showed that the nurses participated in professional ethical training scored higher on the professional values as compared to not participated nurses. The role of continuous education in learning and instilling professional values dealing with ethical dilemmas as well as organization in reinforcing and developing professional values is obvious. Accordingly, it is essential to provide opportunities for nurses to acquire the professional values so that these values improve patient outcomes [36]. Thus, it is suggested that hospitals continually educate their nurses and hold workshops relevant to professional values in order to promote professional nursing.

This study had two limitations. First, the participants’ ranking of the values may be influenced by their degree of understanding of these values. In addition, the nurses’ greater tendency to select the high scores of the Likert scale could be subject to personal bias and exaggeration of scores. To increase the reliability of the findings, a triangulation in data collection such as interviews, observation and a mixed methods study can be helpful. It is also suggested that more accurate instruments be designed to investigate nurses’ perspective on nursing professional values. Second, the NPVS-R measured the importance of professional values from the nurses’ perspective, but it did not assess the application of these values in nursing practice. Therefore, it is necessary to conduct combined studies in the future in order to design instruments to evaluate the application of professional values in nursing practice as well as the predictive factors affecting the use of professional values among nurses.

## Conclusion

In this study, the mean total score of the professional values from the nurses’ perspective was high. The professional values that were directly related to the nursing care and profession were more important. On the other hand, a number of professional values related to non-clinical duties of the nurses such as awareness about role of professional nursing associations and participating in nursing researches were less important. Therefore, the role of healthcare administrators to support less-important professional values and providing the organizational infrastructures in order to increase the nurses’ awareness as well as understanding of the importance of these professional values are emphasized. It is suggested that continuous education programs be designated according to the emerging ethical challenges. Such programs can be a positive step in applying professional values in the nursing practice. As a result, these strategies would promote the nursing profession.

## Abbreviation

NPVS-R: Nursing Professional values scale-revised
